# Herring roe oil in treatment of psoriasis – influence on immune cells and cytokine network

**DOI:** 10.3389/fimmu.2023.1128986

**Published:** 2023-09-08

**Authors:** Aleksandra Petrovic, Ingvild Bueide, Kåre Steinar Tveit, Hogne Hallaråker, Bodil Bjørndal, Tim D. Holmes, Richard Davies, Karl Albert Brokstad, Brith Bergum, Silke Appel

**Affiliations:** ^1^ Broegelmann Research Laboratory, Department of Clinical Science, University of Bergen, Bergen, Norway; ^2^ Department of Dermatology, Haukeland University Hospital, Bergen, Norway; ^3^ Arctic Nutrition AS, Ørsta, Norway; ^4^ Department of Sport, Food and Natural Sciences, Western Norway University of Applied Sciences, Bergen, Norway; ^5^ Core Facility for Flow Cytometry, Department of Clinical Science, University of Bergen, Bergen, Norway

**Keywords:** cellular immune responses, cytokines, polyunsaturated fatty acids, psoriasis, herring roe oil

## Abstract

**Background:**

Psoriasis is a chronic immune-mediated skin disease with systemic inflammation and comorbidities. Although the disease severity may vary over time, many patients suffer from mild to moderate disease. Often local treatment will be sufficient to control the symptoms, but they may have several side effects. ω-3 polyunsaturated fatty acids have shown promising results in clinical trials with mild-to-moderate psoriasis.

**Methods:**

We explored the impact of phospholipid bound docosahexaenoic acid and eicosapentaenoic acid in a 3:1 ratio on immune cells and cytokine networks in peripheral blood of patients with psoriasis. We investigated the inter-relation of plasma cytokine levels and disease severity in 58 patients, and explored the status of circulating immune cell activity in 18 patients with non-severe psoriasis before and during herring roe oil supplementation. Plasma concentration of 22 cytokines was measured by Luminex technology and circulating immune cells were analyzed by multicolor flow cytometry.

**Results:**

CCL2 levels decreased over time, and IFN-γR1 increased, possibly related to the action of ω-3 polyunsaturated fatty acids. We observed a shift from naïve to effector CD4^+^ T cells and decreases of CD38 expression on CD4^+^ and CD8^+^ T cells, CD56^bright^ NK cells and CD14^+^CD16^-^ classical monocytes.

**Conclusions:**

These findings support the beneficial effect of herring roe oil supplementation.

## Introduction

Psoriasis is a complex, lifelong, immune-mediated inflammatory disease of the skin, associated with other medical conditions including psoriatic arthritis, cardiometabolic diseases, chronic kidney disease, gastrointestinal diseases, and psychosocial disorders (1). More than 125 million individuals are affected worldwide, making psoriasis a serious global health problem (2). Disease affects males and females equally and can appear at any age (3). Based on the genetic and immunological characteristics, disease occurs bimodally, before the age of 40 - early onset (type I), and after the age of 40 - late onset (type II). Patients with early onset of psoriasis generally have a family history and more severe disease (4). Psoriasis is usually divided into mild, moderate, and severe based on measurements made with common tools such as body surface area (BSA) and Dermatology Life Quality Index (DLQI), and mild or moderate to severe based on Psoriasis Area and Severity Index (PASI) and DLQI (5). Approximately 80% of the patients have mild disease (6). The severity of psoriasis can fluctuate over time, and symptoms of the disease are usually controlled with treatment, but not always completely. While systemic treatment with biologics has revolutionized the standard of care for patients with severe psoriasis, for most patients with mild to moderate disease, few new treatments have been developed in recent years. Therefore, topical treatment remains the standard, however, due to a variety of side effects, many patients are dissatisfied with their therapy, contributing to low medication adherence (7, 8). Being a life-long disease, novel treatment options are therefore warranted.

Complementary nutritional health approaches have been used in treatment of psoriasis for decades (9). Fish and seafood contain high levels of omega-3 polyunsaturated fatty acids (ω3-PUFA) which have immunomodulatory properties (10). The long chain ω3-PUFAs, eicosapentaenoic acid (EPA) and docosahexaenoic acid (DHA), display therapeutic effects through different mechanisms, including changes in cell membrane lipid composition, cellular metabolism, and signal transduction. They interfere with the production of partially active prostaglandins, thromboxanes without pro-aggregate activity, and leukotrienes of lower chemotactic properties (11, 12). It is thought that they are involved in generation of specialized pro-resolving mediators (SPMs) that are involved in self-regulation of inflammation through switching proinflammatory M1 macrophage phenotype into M2 anti-inflammatory phenotype, recruiting macrophages that can ingest and clean up cellular debris resulting from inflammation, and switching from leukotriene LTB4 to lipoxin 4 which blocks influx of blood neutrophils into tissue (13). Furthermore, SPMs can directly bind to the fatty acid receptor GPR32 to prevent differentiation of T cells into inflammatory subtypes (14). In addition, they can shift pro-inflammatory Th1 responses to Th2 responses through the induction of anti-inflammatory cytokines such as IL-4 and IL-10 and reduced secretion of interferon-γ (14). Further, long-chain ω3-PUFAs affect properties and functionality of the cell membrane by interfering with lipid rafts (15). They downregulate dimerization of toll-like receptors 2 and 4, and reduce trafficking of T cells and their presence in the site of inflammation (16, 17).

A number of clinical studies have been carried out investigating the effect of ω3-PUFAs in psoriasis. So far, one systematic review and one meta-analyses have been published. The results of the systemic review were inconclusive in terms of improvement of PASI score, erythema, scaling, itching, and infiltration (18). The results of meta-analyses based on 10 randomized control trials showed significant reductions in PASI score, erythema, and scaling (19). In our recent investigation, significant improvement of PASI score in psoriasis patients treated with HRO confirmed the beneficial effect of EPA and DHA after 26 and 65 weeks of supplementation (20, 21).

Few studies on animal models of psoriasis have been conducted to investigate the effect of ω3-PUFAs on immune cells, cytokines, and associated responses. Results show decreased production of IL-17, IL-22, IL-23 by Th17 cells, and increased production of Foxp3 by Treg cells as well as inhibition of the migration of skin DCs and IL-17 producing cells (22, 23). Moreover, decrease in STAT3 signaling with reduced responsiveness to IL-6 and subsequent suppression of Th17 cell differentiation has been observed (24). Less is known in humans. It has been shown that dietary intake of fish oil significantly suppress chemotaxis of neutrophils due to decreased LTB4 synthesis, but without proportional clinical improvement of skin lesions in psoriasis patients (25). Consumption of very long-chain ω3-PUFAs may downregulate the expression of CD25 on T cells and thus participate in the anti-inflammatory effect of these fatty acids (26).

The aim of the present study was to investigate the interrelation of inflammatory cytokine levels in plasma and severity of the disease in patients with mild psoriasis (Psoriasis Area Severity Index - PASI < 10) before and during HRO supplementation and correlate to changes in circulating immune cells.

## Materials and methods

### Study design and study population

This immunological investigation as a part of the PSORAX 35 study was undertaken to evaluate effects of HRO on immune cells and cytokines in patients with stable, plaque psoriasis.

Sixty-four individuals, with mild disease (PASI<10), were enrolled in a randomized, placebo-controlled, double blind, 26-weeks long study at the Department of Dermatology, Haukeland University Hospital (20). Each HRO capsule contained 292 mg poly-unsaturated fatty acids (total ω-3): 22% eicosapentaenoic acid (EPA, 20:5 ω-3) and 66% docosahexaenoic acid (DHA, 22:6 ω-3), where approximately 35% of both was bound to phospholipids, including phosphatidylcholine. Each placebo capsule contained medium chain triglycerides: Coconut oil high in caprylic acid (C8:0) and capric acid (C10:0). The same type 590 mg softgel capsule was filled with active substance or placebo (20). Patients were randomly divided into two groups, HRO or control, with patients orally treated with a soft-gel capsule containing active substance, omega-3 polyunsaturated phospholipids (EPA and DHA, 1:3) or a control substance, coconut oil (caprylic acid and capric acid), respectively. The total daily dose of lipids was 5.9 g, of which 2.6 g was active substance.

After 26 weeks, 58 patients (28 patients from active treatment group and 30 patients from control group) continued to participate in a 39-week open-label study receiving only active treatment (21).

For our investigation, we included 58 patients from the PSORAX 35 study to explore plasma cytokine levels at four time points (inclusion, after 12, 26, and 65 weeks of treatment). Next, to investigate immune cells and their activation status at inclusion and after 26 weeks of treatment, we selected 18 psoriasis patients, 10 from HRO group and 8 from control group. The following inclusion criteria were used: early onset of the disease, absence of psoriatic arthritis, and non-use of immunomodulatory treatment and drugs that affect lipid metabolism such as cyclosporine, tacrolimus, corticosteroids, and antiviral therapy.

The study was approved by the regional ethics committee (approvals 2017/938). Written informed consent was obtained from all participants. An overview of the patients’ characteristics is shown in **Table 1**.

**Table 1:** Characteristics of patients for cytokine levels analysis (n = 58) and immune cells investigation (n = 18) at inclusion and follow-up, approximately 12, 26, and 65 weeks after starting HRO treatment.

Sex (M/F)		Age	Onset of P < 40	Onset of P ≥ 40	AR	BMI	PASI w 0	PASI w 12	PASI w 26	PASI> w 65
Patients for cytokine levels analysis										
HRO	17/12	46.62 (13.42)	23 (n) 20.87 (9.20)	6 (n) 54.17 (14.63)	14	29.88 (5.88)	6.22 (1.90)	5.92 (2.74)	4.23 (2.49)	2.78 (1.8)
Control	18/11	52.07 (13.75)	24 (n) 21.37 (10.12)	5 (n) 49 (8.69)	14	28.83 (3.88)	5.99 (1.75)	5.48 (2.53)	5.48 (2.58)	2.96 (2.1)
Patients for immune cells investigation										
HRO	5/5	45,64 (13,38)	17 (8.16)	0	0	29.95 (6.54)	5.75 (1.42)	6.15 (2.30)	5.19 (2.56)	3.36 (1.6)
Control	6/2	45.37 (11.03)	20.12 (9.85)	0	0	31.42 (4.76)	6.36 (1.75)	5.71 (2.18)	5.64 (2.83)	3.25 (1.9)

AR, arthritis; BMI, body mass index; PASI, Psoriasis Area Severity Index. Values are listed as mean (SD).

### Blood sampling

All samples were collected at the Research Unit for Health Surveys, Department of Clinical Science, University of Bergen, Norway. Plasma samples were collected at week 0, 12, 26 and 65 weeks and stored at –80° C until use.

Peripheral blood samples were collected in a Vacutainer^®^ CPT™ tube (BD) at the Department of Dermatology, Haukeland University Hospital, Bergen, Norway. Peripheral blood mononuclear cells (PBMC) were isolated within one hour following manufacturer’s instructions, cryopreserved in fetal calf serum (FCS) containing 10% dimethyl sulfoxide (DMSO) and stored at – 150°C until use.

### Flow cytometry

Twelve fluorochrome-conjugated antibodies and one dye, Pacific orange (PO), were used to identify and analyze activity state of the main mononuclear immune cell types, T cells, monocytes, B cells, NK cells and NKT-cells. PO dye was used as a live/dead marker (**Supplementary Table 1**).

Before staining, cryopreserved PBMC samples were rapidly thawed using a water bath set to 37°C then washed once in prewarmed X-vivo 20™ containing Nuclease (1:10,000; Pierce™ Universal Nuclease for Cell Lysis; Thermo Fisher Scientific, MA, USA) and centrifuged at 453 x g for 5 min at 23°C. The cells were resuspended in prewarmed X-vivo 20™ and counted with a CASY cell counter (Schärfe System) and washed in cold Phosphate Buffered Saline (PBS). After resuspending in cold PBS, up to 5 million cells were stained with Pacific Orange (1:1000, stock concentration: 0.5 mg/ml, Thermo Scientific) for 30 min on ice in the dark. Cells were then washed once and resuspended in FACS buffer (PBS + 0.5% BSA) containing 2µl FcR block (Miltenyi Biotec, Bergisch Gladbach, Germany) per 1 × 10^6^ cells for 5 min and stained with the antibody master mix for 30 minutes in the dark at 4°C. After washing, samples were resuspended in fixation buffer 1,6% PFA (1:10, stock PFA 16%, Thermo Fisher) for 12 min at room temperature. The last wash and resuspension in FACS-buffer was followed by analysis of the samples at the flow cytometer. The entire experiment was run in two days, all samples were stained from two antibody master mixes and acquired after staining. Single stained compensation controls were made with OneComp eBeads (eBioscience). Two cryopreserved PBMC samples from one healthy donor were run as internal control for assessing possible technical variations in the two batches of the experiment.

Samples were acquired on a LSRI Fortessa flow cytometer (BD Biosciences, San Jose, CA, USA) with BD FACSDiva™ Software (BD Biosciences) at the Flow Cytometry Core Facility, University of Bergen, Norway. The flow cytometer was equipped with 407, 488, 561, and 635 nm lasers. Further specification is given in **Supplementary Table 2**. The flow cytometer was regularly calibrated with BD cytometer setup and tracking beads (BD Biosciences). Fluorescence minus one (FMO) controls were used for defining the gates. The positive and negative gates were defined based on biaxial plots. An average of 1.175.113 events in the intact cell gate were collected for each sample and mean percentage of live cells was 91%. Flow cytometry data was initially analyzed in FlowJo v10.6.1 (Tree Star, Ashland, OR, USA) and then exported to Microsoft Excel to prepare for further statistical analysis in GraphPad Prism. A representative gating strategy for a single donor is shown in **Supplementary Figure 1**. The surface markers used to define the immune cell subsets are listed in **Supplementary Table 3**.

### Luminex technology

In this study, we used a custom-designed Human Luminex Discovery Assay LXSAHM-20 (CCL2, CCL3, CCL4, CD25/sIL-2Rα, CXCL9, CXCL10, IFN-γ, IFN-γ R1, IL-1α, IL-1β, IL-2, IL-6, IL-8, IL-10, IL-12, IL-17, IL-21, IL-33, TNF-α, VEGF) and LXSAHM-01 (separately CCL5 and IL-23) (R&D systems, McKinley, MN, USA) with some modifications according to the manufacturer’s instructions. The following adjustments were made: i) one additional standard was included in the three-fold serial dilution making the standard range from 1:3 to 1:2.187; (ii) samples were diluted 1:2 for the 20-plex and IL-23, and 1:50 for CCL5. Data was acquired on a Luminex 100 System (Luminex Corp., Austin, TX, USA) and StarStation Software v.3.0 [Applied Cytometry System, Dinnington, UK). The five-parameter logistic algorithm (weighted by 1/y, (V2.4)] and raw median fluorescence intensity values were used for the creation of standard curves.

### Statistical analysis

The Mann-Whitney U test was used in the comparison between the study groups in Luminex and flow cytometry analysis, and Wilcoxon signed rank test for paired samples was performed to study significant differences in PASI and DLQI score between different time points. Spearman’s correlation was applied to find significant interrelation between cytokines and disease severity. Degree of correlation was interpreted according to the recommendation of the British Journal of Medicine (https://www.bmj.com/about-bmj/recources-readers/publications/statistics-square-one/11-correlation-and-regression) with r: 0.00-0.19 (very weak), 0.20-0.39 (weak), 0.40-0.59 (moderate), 0.60-0.79 (strong), 0.80-1.00 (very strong). Differences were considered statistically significant when p <0.05. No correction was made for multiple comparisons since the study was of exploratory nature. The comparisons between patient groups on HRO and coconut oil, and the production of associated graphs and figures were done using GraphPad Prism (Version 9.1.1 (225)).

## Results

### Psoriasis patients on herring roe oil treatment have alterations in peripheral blood immune cells compared to placebo controls

In a randomized, double-blind, placebo-controlled, single center study it was previously shown that psoriasis patients receiving HRO had a significant reduction in mean PASI score from baseline to week 26 compared to the control group (20). Here we followed up these findings and analyzed the composition and phenotype of PBMC in a selection of patients included in the study.

**Figure 1:**
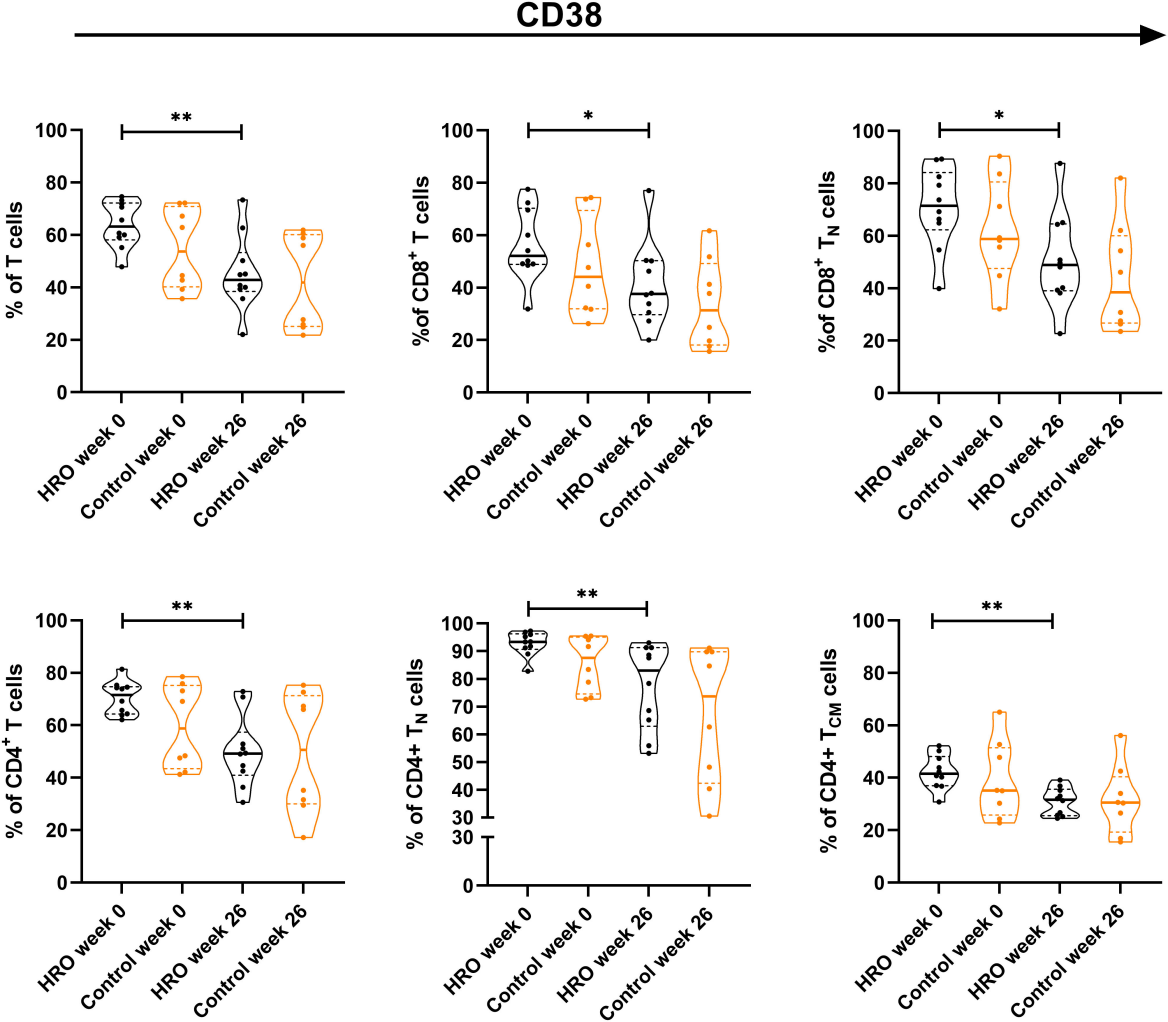
Differences in frequency of CD38^+^ T cells between herring roe oil (HRO) treated and placebo (coconut oil; Control) treated patients with psoriasis during the study period of 26 weeks. HRO treatment (n=10), Control (n=8). Each symbol represents one individual. Violin plot (truncated) shows median and quartiles. Mann-Whitney test was used to compare abundance of CD38^+^ T cell population and subpopulations in both groups. Differences were considered statistically significant for p ≤ 0.05, with significancy be indicated as *≤ 0.05 and **≤ 0.01.

No differences between HRO and the control group in the frequency of analyzed immune cell populations were found, and changes in frequency over time were similar between the groups (**Supplementary Figure 2**). However, we found a significant reduction of CD38^+^ T cells (p=0.0068) in HRO treated patients after 26 weeks, both CD4^+^ (p=0.0015) and CD8^+^ T cells (p=0.0373), whereas the control group did not reach statistical significance. Further analyses revealed reduction of CD38^+^ CD4^+^ T_N_ cells (p=0.0026), CD4^+^ T_CM_ cells (p=0.0011) and CD8^+^ T_N_ cells (p=0.0115) in patients treated with HRO (**Figure 1**). Only small alterations in expression of activation markers HLA-DR and CD69 in T cells was observed (**Supplementary Figure 3**).

**Figure 2:**
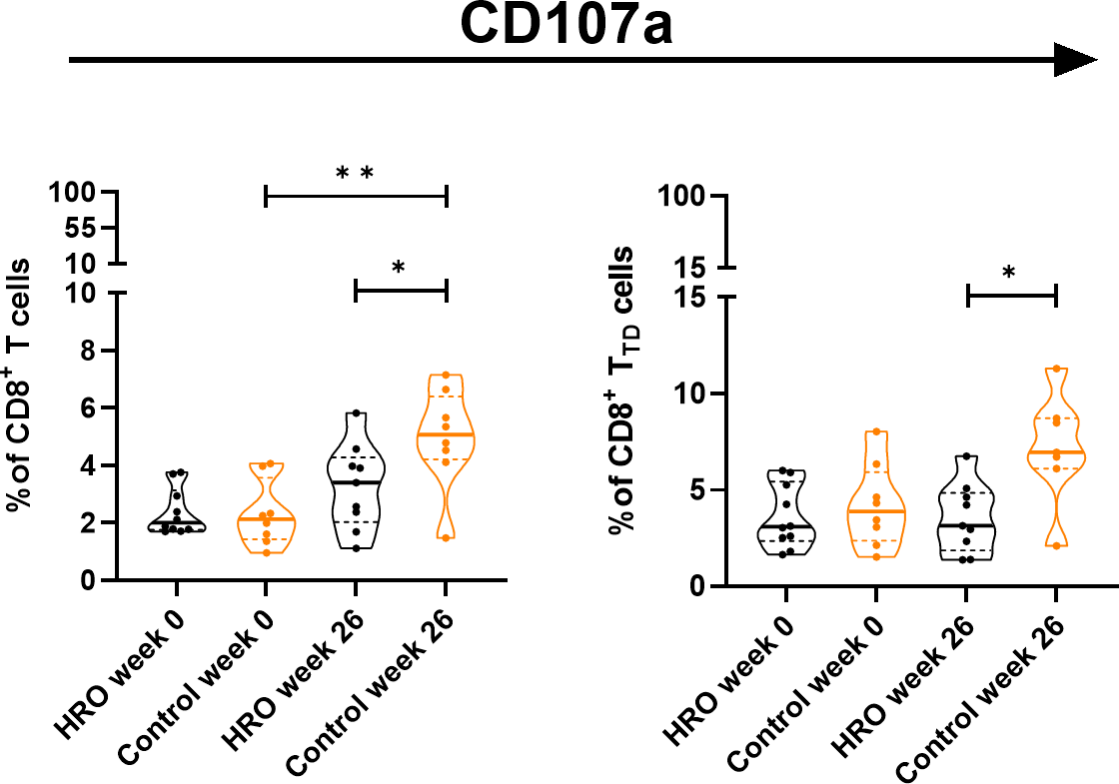
Differences in frequency of CD107a^+^ T cell population and CD107a^+^CD8^+^ T cell subsets between HRO and control group during the study period of 26 weeks. HRO treatment (n=10), Control (n=8). Each symbol represents one individual. Violin plot (truncated) shows median and quartiles. Mann-Whitney test was used to compare abundance of T cell population and subpopulations in both groups. Differences were considered statistically significant for p ≤ 0.05, with significancy be indicated as *≤ 0.05, **≤ 0.01.

**Figure 3:**
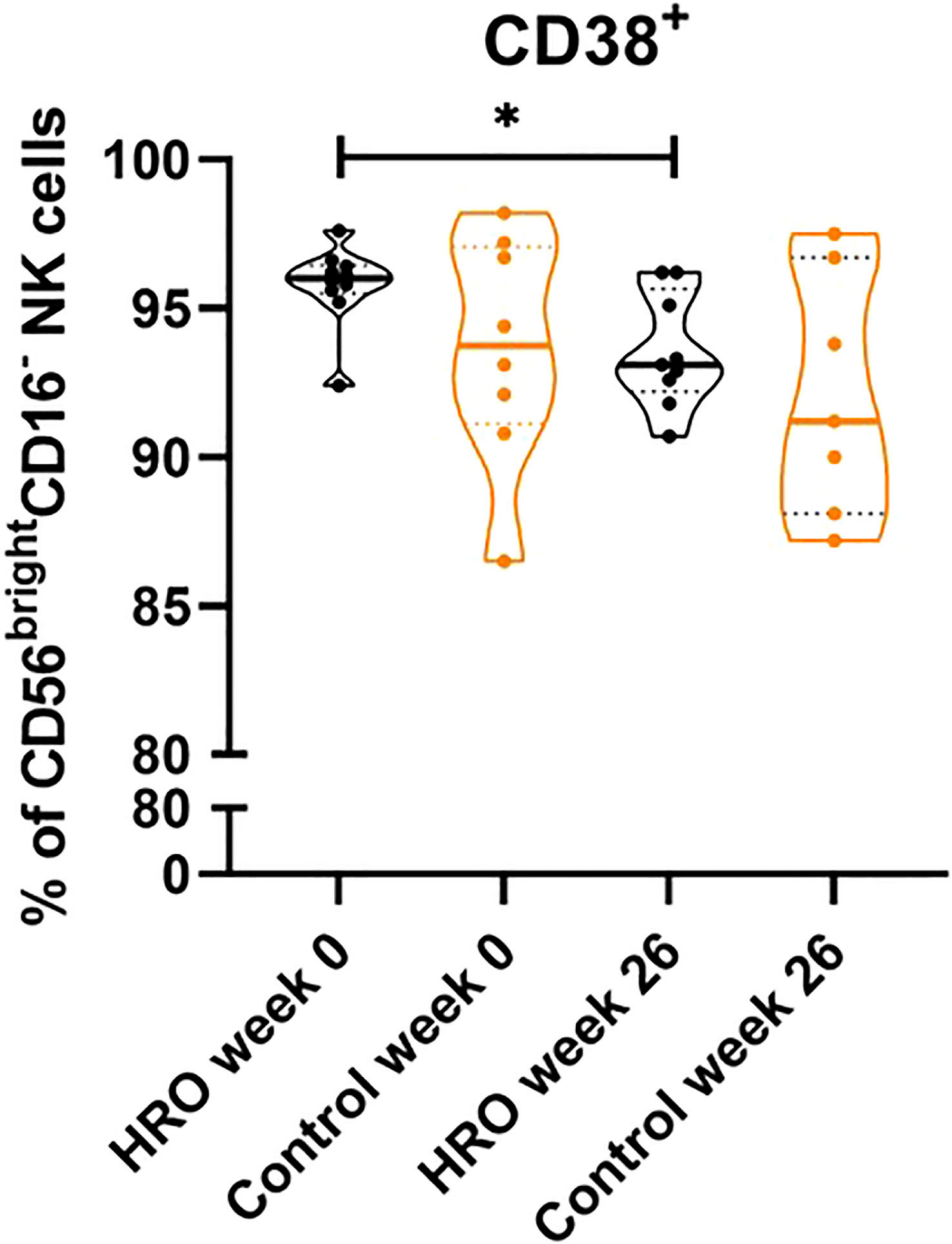
Reduction of CD38 expression on CD56^bright^CD16^-^ NK cells upon treatment with HRO. The data represents HRO patients (n=10) and Control group (n=8) measured in different time points (week 0 and 26). Each symbol represents one individual. Violin plot (truncated) shows median and quartiles. Mann-Whitney test was used to compare abundance of subsets of PBMCs in both groups. Differences were considered statistically significant for p ≤ 0.05, with significancy be indicated as *≤ 0.05.

In addition, we observed a significantly higher percentage of CD107a^+^CD8^+^ T cells (p=0.0464), and CD107a^+^CD8^+^ T_TD_ cells (p=0.0164) in the control group compared to HRO treated patients (**Figure 2**). A similar pattern was also observed in NK cells (**Supplementary Figure 4**). We next determined expression of other activation markers on peripheral NK cells and observed a significant reduction of CD38^+^ CD56^bright^CD16^-^ NK cells after treatment with HRO (p=0.0276; **Figure 3**).

**Figure 4:**
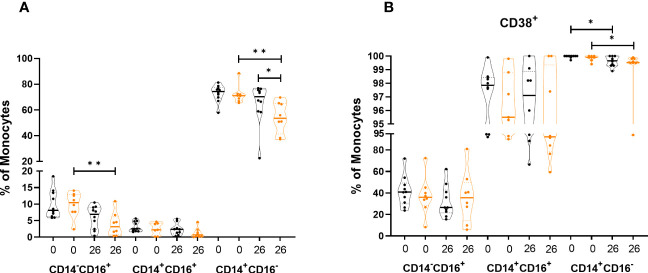
Differences in frequency and expression of activation markers in monocytes between herring roe oil (HRO) treated and placebo (coconut oil; Control) treated patients with psoriasis during the study period of 26 weeks. **(A)** Frequency of monocyte subpopulations. **(B)** Expression of activation marker CD38 on monocyte subpopulations. The data represents HRO patients (n=10) and Control group (n=8) measured in different time points (week 0 and 26). Each symbol represents one individual. Violin plot (truncated) shows median and quartiles. Mann-Whitney test was used to compare abundance of subsets of PBMCs in both groups. Differences were considered statistically significant for p ≤ 0.05, with significancy be indicated as *≤ 0.05, **≤ 0.01.

We then analyzed frequencies of the different monocyte populations. The frequency of classical monocytes in the controls was significantly lower (p=0.0343) compared to the HRO group at week 26 (**Figure 4A**). Analysis of activation markers on classical monocytes showed a significant decrease in expression of CD38 over time in both patient groups (HRO p=0.0108; Control p=0.0297) (**Figure 4B**).

Assessment of technical variations by analysis of two internal controls showed low inter-assay variability. Coefficient of variation was less than 15% in parent populations (T cells 9.5%, B cells 2.5%, NK cells 14.9%, monocytes 1.9%, and NKT-like cells 5.8%).

### Changes of cytokine levels during long-term supplementation with herring roe oil

**Figure 5:**
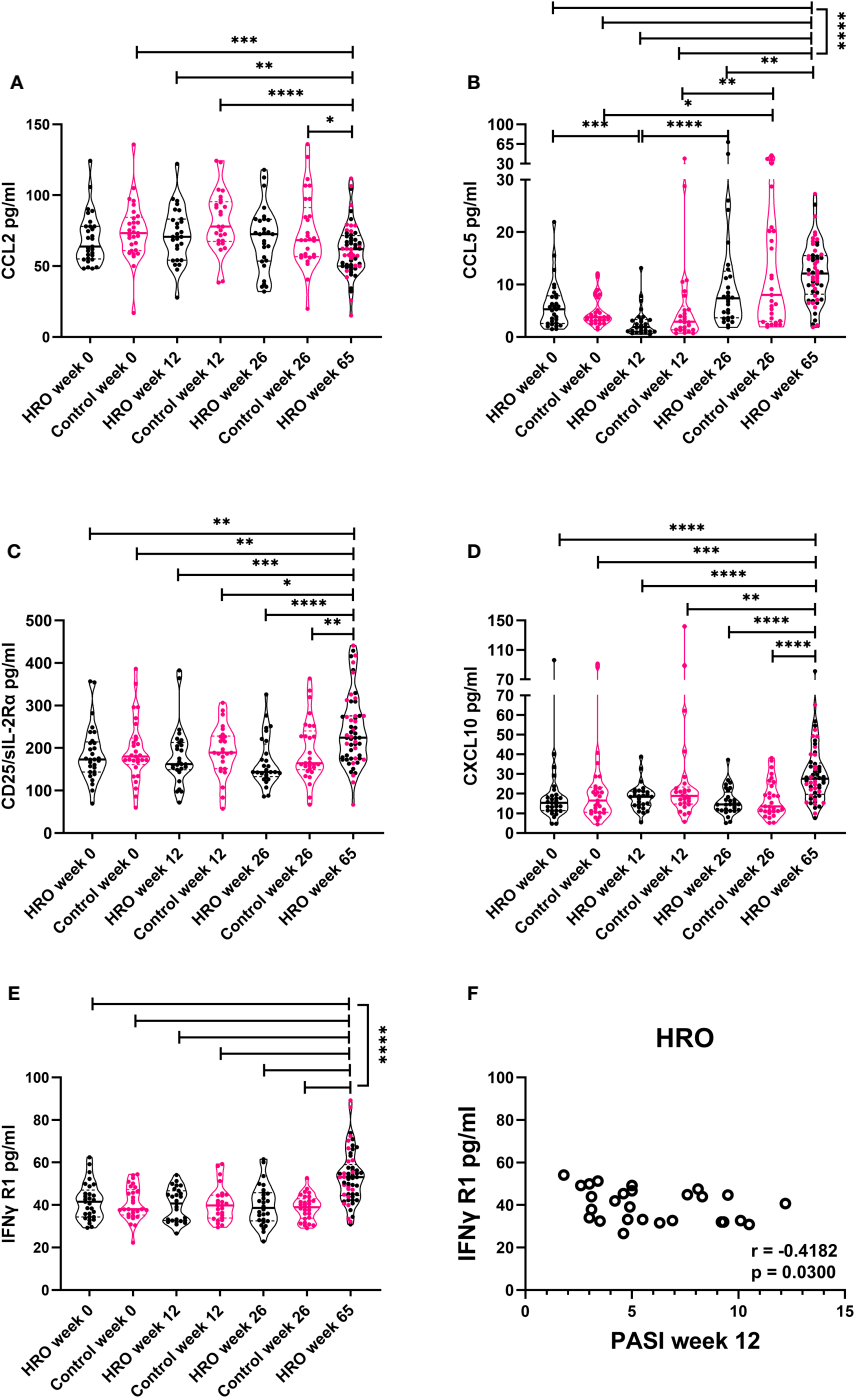
Changes of cytokine levels during long-term supplementation with herring roe oil. Cytokine profiles in plasma were measured utilizing Luminex technology. Plasma levels of **(A)** CCL2, **(B)** CCL5, **(C)** CXCL10 **(D)** CD25/sIL-2Rα, and **(E)** IFN-γ R1 in HRO week 0 (n=29), HRO week 12 (n=27), HRO week 26 (n=28), HRO week 65 (n=57), Control week 0 (n=29), Control week 12 (n=26), and Control week 26 (n=29).Comparison between pairs was done by unpaired Mann-Whitney test between the patients and controls. Differences were considered statistically significant for p ≤ 0.05, with significancy indicated as *≤ 0.05, **≤ 0.01, ***≤ 0.001, ****≤ 0.0001. **(F)** Correlation between IFN-γ R1 level and PASI score at week 12 in patients supplemented with herring roe oil.

We next analyzed levels of several cytokines in 57 patients who had continued for an additional 39 weeks on HRO after the end of the 26-week randomized trial with beneficial clinical effects (20) (**Figure 5**). This effect represented by a decrease in PASI score had been obvious also in the 65th week of the study (21). Five of the analytes were detected in 100% of samples (CCL5, CCL2, CD25/sIL-2Rα, CXCL10 and IFN-γ R1), whereas others were only detected in 25-45% (IL-8 and VEGF) and <25% of samples (CCL4, IFN-γ, IL-1α, CCL3, IL-6, IL-10, IL-23, IL-33, IL-17, IL-21, TNF-α, CXCL9, and IL-1β). IL-2 and IL-12 were below detection limit in all samples. CCL2 decreased over time from week 12 in both treatment groups and was significantly decreased at week 65 (**Figure 5A**). In contrast, CCL5, CD25/sIL-2R, CXCL10, and IFN-γR1 levels increased at the end of the study period (**Figure 5E**). No significant differences between HRO and control group were detected.

Correlation analysis of the individual cytokines of the two groups at different time points to disease severity (PASI) revealed moderate negative correlation (r=-0.4182; p=0.03) between PASI score at week 12 and IFN-γ R1 plasma levels in patients treated with HRO (**Figure 5F**).

## Discussion

A number of studies have evaluated the clinical impact of dietary supplementation with ω3-PUFAs, particularly EPA and DHA, in limiting and repairing of skin inflammation (27). Due to the anti-inflammatory properties of ω3-PUFAs, their oral supplementation is a useful monotherapy, and conjunctive therapy improving the effects of topical, oral and phototherapy in psoriasis patients (19, 28–30). However, only few reports have investigated the effect of these nutrients on the immune cell profile in patients with psoriasis (25, 26). Our patients on stable local anti-psoriatic maintenance treatment showed improvement of PASI score after 26- and 65-week long supplementation with HRO (20, 21). We therefore analyzed its impact on peripheral blood immune cells and found a decrease in CD38 expression on CD4^+^ and CD8^+^ T cells, CD56^bright^ NK cells and CD14^+^CD16^-^ classical monocytes supporting the beneficial effect of HRO supplementation on immune cell activation. Regarding the plasma cytokine levels, the decrease in chemokine CCL2 and increase of IFN-γR1 could be related to the action of herring roe oil/ω3-PUFAs.

We observed a shift from naïve to effector CD4^+^ T cells in patients treated with HRO not seen in patients receiving placebo. This is in accordance with our previous study showing that patients with severe disease treated with various biopharmaceuticals (anti-TNF, anti-IL-17 or anti-IL-12/23 treatment) restore and equalize the amount of CD45RO^+^ CD4^+^ T cells (31). However, HRO supplementation was not accompanied by a reduction in CD8^+^ T cells as described in the same study (31). One possible explanation for this discrepancy might be the differences in disease severity.

Exploring activity state of T cells, CD38 and HLA-DR are of particular interest in psoriasis patients. Significant evidence indicate CD38 plays a significant role in inflammation and autoimmunity, where CD38 plays roles in modulating cell differentiation and effector functions (32). CD38 can establish lateral associations with various membrane proteins/complexes, including CD3/TCR, modulating activation thresholds from these complexes. Lipid rafts play an important role in the stability and function of such complexes (33). Long-chain ω3-PUFAs could potentially interfere with signaling proteins in lipid rafts of the cell membrane, for which we were particularly interested in their effect on CD38 expression (15). In our study, patients treated with HRO showed decreased expression of CD38 in both CD4^+^ and CD8^+^ T cells, indicating a decreased activity of these cells. Therefore, further investigation of CD3/TCR activation thresholds would be of interest.

Few studies have investigated CD107a/lysosomal-associated membrane protein 1 (LAMP-1) in psoriasis. Interestingly, our patients treated with coconut oil had an increase in frequency of CD107a^+^ CD8^+^ T cells over time, suggesting their higher activity/cytotoxic potential, even though without clinical deterioration (34). To date, no human studies (clinical or observational) have assessed potential preventative or therapeutic effects of orally administered coconut oil in patients with psoriasis.

In active psoriasis, decreased frequency of CD56^bright^CD16^−^ and CD56^dim^CD16^+^ NK cells in circulation has been observed, probably as the result of chemokine-dependent recruitment of these cells from the peripheral blood to inflamed skin (35–37). In a study conducted in patients with psoriasis and atopic dermatitis, reductions of CD56^bright^ NK cells were connected with the previous findings of accumulated CXCR3 expressing CD56^bright^ NK cells in lesional skin, probably due to CXCL10 production by psoriatic keratinocytes (38). In our study, stable plasma levels of CXCL10 chemokine in both patient groups during the 26 weeks could support steady frequencies of circulatory CD56^bright^ NK cells. Furthermore, during the HRO supplementation these cells showed lower expression of CD38 surface molecule indicating possible reduced capacity in transduction of activating signals.

Several studies have confirmed association of psoriasis to cardio-vascular diseases and metabolic syndrome (1, 39, 40). Circulating monocytes are important cellular players in the development and progression of both diseases. In peripheral blood of patients with psoriasis, elevated levels of CD14^+^CD16^+^ intermediate monocytes and reduced levels of CD14^+^CD16^-^ classical monocytes have been observed (31, 41). These two subsets of monocytes act as a proatherogenic cells while CD14^-^CD16^+^ non-classical monocytes exert an atheroprotective effect as they maintain vascular homeostasis (42). In our study, HRO treatment did not affect frequency of monocyte subsets. However, we observed a decrease in CD14^-^CD16^+^ non-classical monocytes in the control patients treated with coconut oil indicating a higher risk of developing atherosclerosis in these obese patients (40). At the same time, the observed decrease of classical monocytes might reduce the risk of atherosclerosis. This needs further investigations and a follow-up over time.

A modest number of studies have been conducted to investigate the impact of ω3-PUFAs to the mediators of inflammation in psoriasis. In animal models, reduced production of proinflammatory cytokines IL-17, IL-22, and IL-23 have demonstrated the anti-inflammatory effect of n-3 polyunsaturated fatty acids and their bioactive metabolite (22, 23, 43). To date, no investigation of the impact of HRO on cytokines in patients with psoriasis has been done. In our study, not all analyzed cytokines were detected, which was expected in patients with mild disease.

The ligand-binding IFN-γR1 chains together with signal-transducing IFN-γR2 chains are responsible for forming the IFN-γ receptor that binds to IFN-γ. The result of signal transduction is an intracellular signaling network activation mainly via the JAK/STAT pathway, which modulates the transcription of hundreds of genes and mediates diverse biological responses, including proliferation and auto-control of the IFN-γ receptor expression (44, 45). We observed a negative moderate correlation between the lowest level of IFN-γR1 measured at week 12 and PASI score in patients treated with HRO. One might speculate that the supplementation with HRO caused the disturbance in signal transduction through the IFN-γ receptor with the consequent beneficial effect on disease severity.

Elevated levels of serum/plasma sIL-2Rα (CD25) has been observed in individuals with various immune-mediated diseases including psoriasis (46). Moreover, its serum concentration as well as the frequency of CD25^+^ cells in lesional skin correlate with disease severity expressed by PASI. We observed a significant increase of sIL-2Rα following treatment with HRO despite the clear improvement in PASI which needs further analysis.

The binding of CCL2 to the chemokine receptor CCR2, mainly expressed on the surface of monocytes, induces migration from the circulation into lesional skin and differentiation into macrophages. These cells are able to act as antigen-presenting cells and secrete TNF-α responsible for maintaining skin inflammation (47). It has also been observed that ω3–PUFAs consumption inversely correlates with the levels of peripheral C3 and CRP concentration (48). Elevated levels of circulatory CCL2 in patients with psoriasis have been observed in several studies (49, 50). Various therapeutic modalities such as anti-TNF, anti-CD11, combination of coal tar and UV light, and narrow-band ultraviolet B (NB-UVB) phototherapy induce the decrease of CCL2 in the circulation (49, 51, 52). We here show that HRO supplementation has a similar effect.

A limitation of the current study is the lack of PBMC samples at week 65, lack of a healthy control population as well as lack of skin biopsy specimens of psoriatic lesions.

Treatment of psoriasis patients has improved with introduction of systemic biopharmaceuticals over the last decades. However, for more than 80% patients with mild to moderate disease the topical treatment remains the standard, but with considerable side effects (7). Therefore, the use of HRO as a supplementary treatment option in patients with mild psoriasis is a promising treatment option for this patient group. Future studies exploring simultaneously immune cells in circulation and skin biopsy specimens in a larger number of psoriasis patients and healthy controls might shed further light on the utility of HRO.

## Data availability statement

The data that support the findings of this study are available on request from the corresponding author. The data are not publicly available due to privacy or ethical restrictions.

## Ethics statement

The studies involving humans were approved by Western Norway ethical committee (approval 2017/938). The studies were conducted in accordance with the local legislation and institutional requirements. The participants provided their written informed consent to participate in this study.

## Author contributions

SA, KAB, AP, KST, HH, BoB, BrB, TDH and RD contributed to conception and design of the study. AP and IB performed experiments. AP and IB analyzed the data. AP wrote the first draft of the manuscript. All authors contributed to manuscript revision, read, and approved the submitted version.
