# Supplementary material for: Herring roe oil in treatment of psoriasis – influence on immune cells and cytokine network
Source: Front Immunol. 2023 Sep 8;14:1128986. doi: 10.3389/fimmu.2023.1128986 (PMC10515196; doi:10.3389/fimmu.2023.1128986)
Supplement: Supplementary file 1 [file DataSheet_1.docx]

**Supplementary material**

**Suppl. Table 1**. Listing of applied fluorochrome labelled monoclonal antibodies

| Marker | Fluorochrome | Clone | Dilution | Producer |
| --- | --- | --- | --- | --- |
| CD19 | AF488 | HIB19 | 1:400 | BioLegend |
| CD8 | PerCP-Cy5.5 | RPA-T8 | 1:400 | BioLegend |
| CD38 | PE | HIT2 | 1:80 | BioLegend |
| CD69 | PE-Cy7 | FN50 | 1:80 | BioLegend |
| HLA-DR | PE-TR/ECD | G46-6 | 1:640 | BD Biosciences |
| CD4 | APC-Cy7 | RPA-T4 | 1:400 | BioLegend |
| CD3 | AF700 | UCHT1 | 1:40 | BioLegend |
| CD56 | AF647 | HCD56 | 1:80 | BioLegend |
| CD45RO | BV785 | UCHL1 | 1:80 | BioLegend |
| CD14 | BV605 | M5E2 | 1:80 | BioLegend |
| CD27 | BV650 | O323 | 1:40 | BioLegend |
| CD107a | BV421 | H4A3 | 1:80 | BioLegend |

**Suppl. Table 2.** Specification of the LSRI Fortessa flow cytometer

| Fluorochrome | Emission filters |
| --- | --- |
| Alexa Fluor 488 | LP: 505, BP: 530/30 |
| PerCP-Cy5.5 | LP: 685, BP: 695/40 |
| PE | LP: -, BP: 582/15 |
| PE-Cy7 | LP: 750, BP: 780/60 |
| PE-TR/ECD | LP: 600, BP: 610/20 |
| APC-Cy™7 | LP: 750, BP: 780/60 |
| Alexa Fluor-700 | LP: 710, BP: 730/45 |
| AF647 | LP: -, BP: 670/14 |
| BV785 | LP: 750, BP: 780/60 |
| BV605 | LP: 595, BP: 605/12 |
| BV650 | LP: -, BP: 670/30 |
| BV421 | LP: -, BP: 450/50 |
| BV711 | LP: -, BP: 710/40 |
| Pacific orange | LP: 570, BP: 585/42 |

**Suppl. Table 3**. Immune cell subset definitions by cell surface markers.

| Population name | CD molecules |
| --- | --- |
| T cells | CD14^-^CD56^-^CD3^+^ |
| T helper cells | CD14^-^CD56^-^CD3^+^CD4^+^ |
| Naïve | CD14^-^CD56^-^CD3^+^CD4^+^CD45RO^-^CD27^+^ |
| Central memory | CD14^-^CD56^-^CD3^+^CD4^+^CD45RO^+^CD27^+^ |
| Effector memory | CD14^-^CD56^-^CD3^+^CD4^+^CD45RO^+^CD27^-^ |
| Terminally differentiated | CD14^-^CD56^-^CD3^+^CD4^+^CD45RO^-^CD27^-^ |
| T cytotoxic cells | CD14^-^CD56^-^CD3^+^CD8^+^ |
| Naïve | CD14^-^CD56^-^CD3^+^CD8^+^CD45RO^-^CD27^+^ |
| Central memory | CD14^-^CD56^-^CD3^+^CD8^+^CD45RO^+^CD27^+^ |
| Effector memory | CD14^-^CD56^-^CD3^+^CD8^+^CD45RO^+^CD27^-^ |
| Terminally differentiated | CD14^-^CD56^-^CD3^+^CD8^+^CD45RO^-^CD27^-^ |
| NK cells | CD14^-^CD3^-^CD56^+^ |
| Bright | CD14^-^CD3^-^CD56^bright^CD16^-^ |
| Intermediate | CD14^-^CD3^-^CD56^+^CD16^+^ |
| Dim | CD14^-^CD3^-^CD56^dim^CD16^+^ |
| NKT-like cells | CD14^-^CD3^+^CD56^+^ |
| Monocytes | CD56^-^CD3^-^CD19^-^CD14^+/-^ |
| Non-classical | CD56^-^CD3^-^CD19^-^CD14^-^CD16^+^ |
| Intermediate | CD56^-^CD3^-^CD19^-^CD14^+^CD16^+^ |
| Classical | CD56^-^CD3^-^CD19^-^CD14^+^CD16^-^ |
| B cells | CD3^-^CD19^+^ |
| Transitional | CD27^-^CD38^high^ |
| B naïve cells | CD27^-^CD38^+/-^ |
| B memory cells | CD27^+^CD38^+/-^ |
| Plasmablasts | CD27^+^CD38^bright^ |


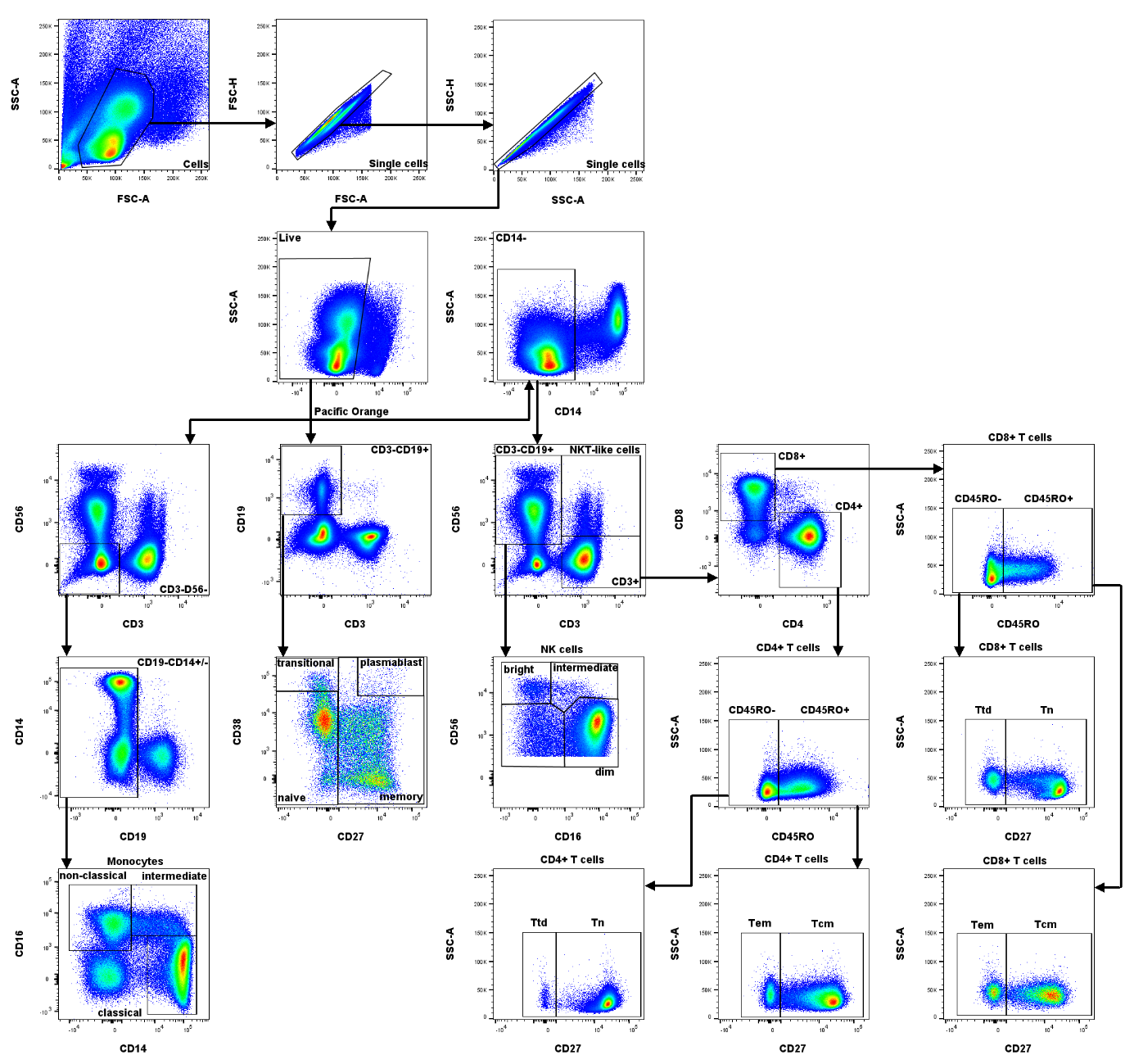


**Suppl. Figure 1.** Representative gating strategy. From the live single cell gate, B cells and monocytes were gated. For analysis of B cells, live single cells were gated on CD3^-^CD19^+^ cells. To identify memory (CD27^+^CD38^+/-^), naïve (CD27^-^CD38^+/-^), transitional (CD27^-^CD38^bright^) B cells, and plasma blasts (CD27^+^CD38^high^), CD38 and CD27 were used. To identify monocytes, live single cells were gated on CD3^-^CD56^-^ and then on CD19^-^CD14^+/-^. Three subtypes of monocyte, classical (CD14^+^, CD16^-^), non-classical (CD14^-^CD16^+^), and intermediate (CD14^+^CD16^+^) were identified. From the CD14^-^ population, both T cells and NK cells were gated on CD3 vs CD56. CD3^+^ T cells were divided into CD8^+^ T cells and CD4^+^ T cells. Each of these populations were further subdivided into four subpopulations based on CD45RO and CD27 expression: CD45RO^-^CD27^+^ naïve (T_N_), CD45RO^+^CD27^+^ central memory (T_CM_), CD45RO^+^CD27^-^ effector memory (T_EM_) and CD45RO^-^CD27^-^ terminally differentiated (T_TD_) T cells. CD3^-^CD56^+^ NK cells were further subdivided into CD56^dim^CD16^+^ and CD56^bright^CD16^-^ NK cells. The double positive CD3^+^CD56^+^ cells were defined as NKT-like cells.


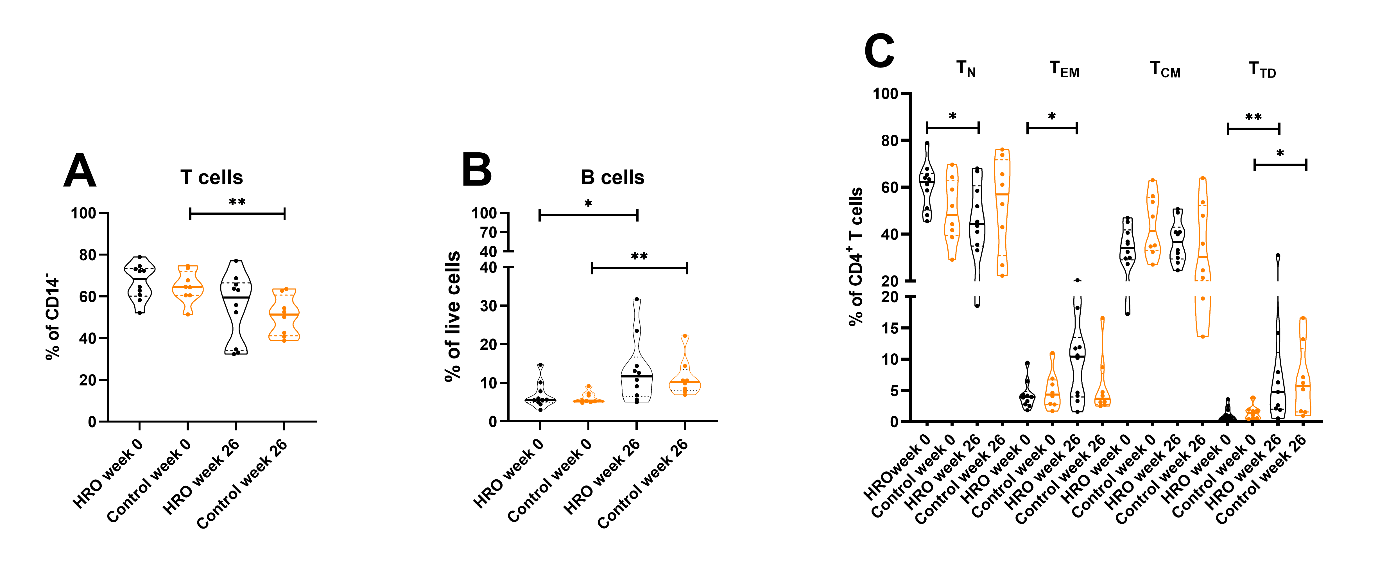


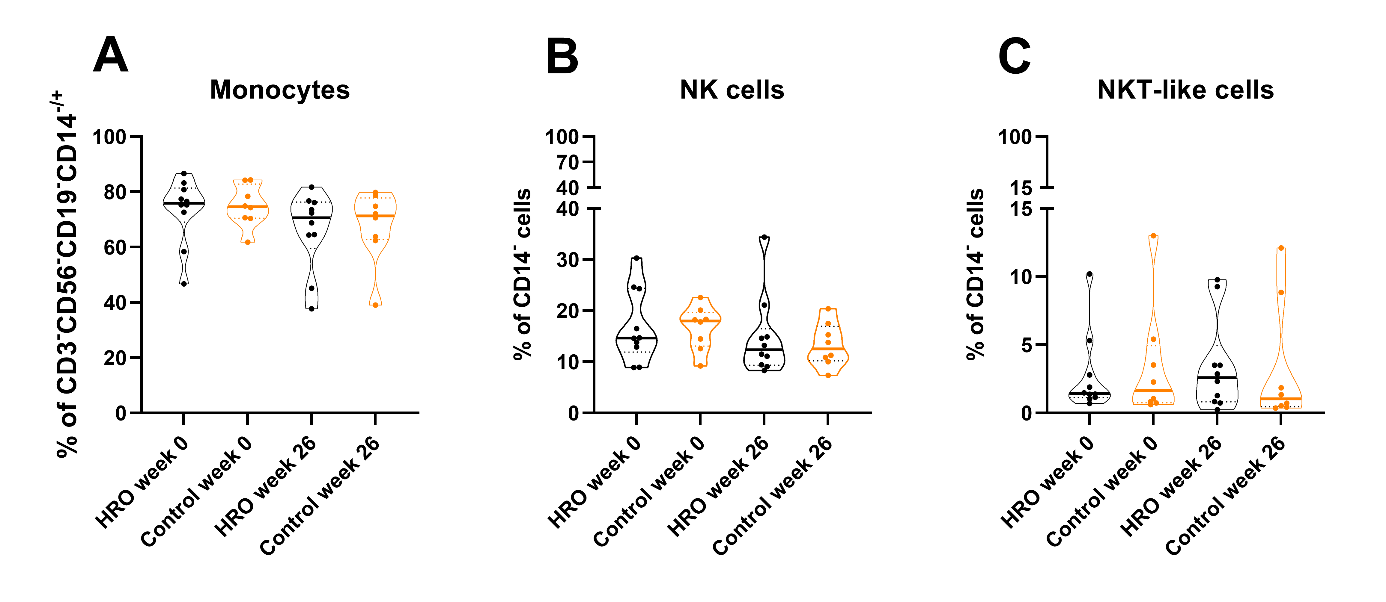
 **D E F**


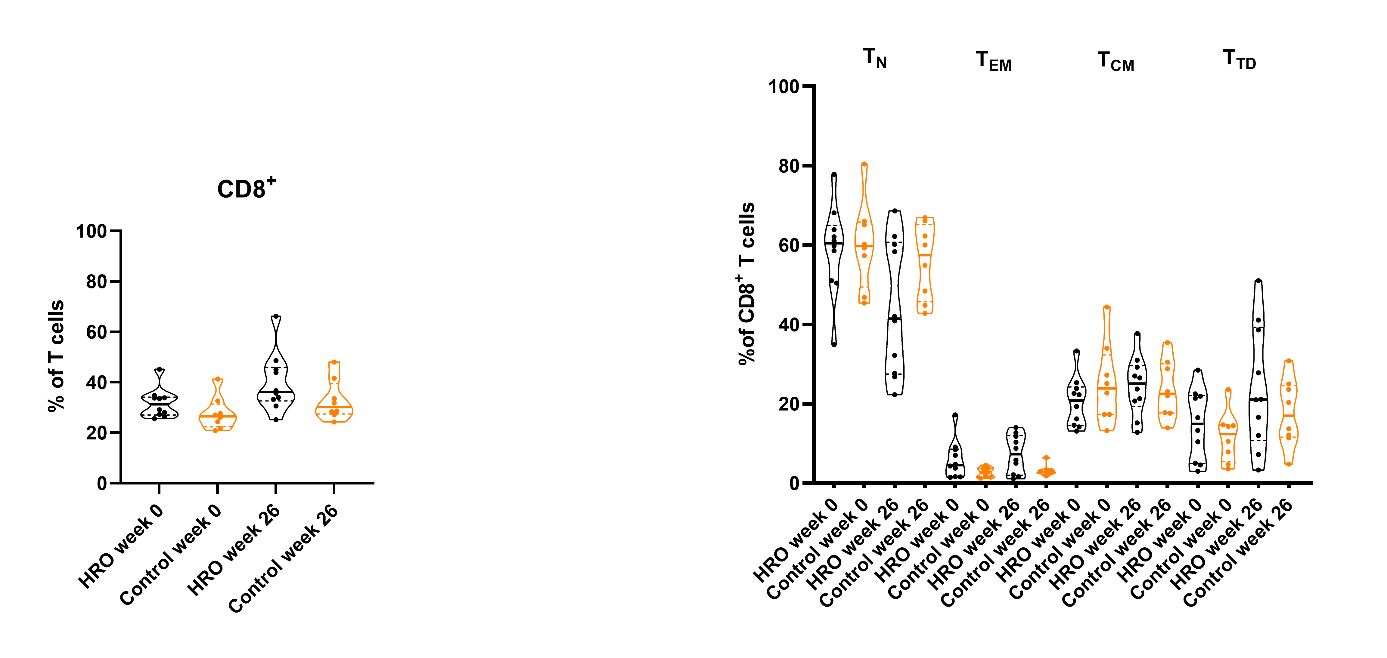

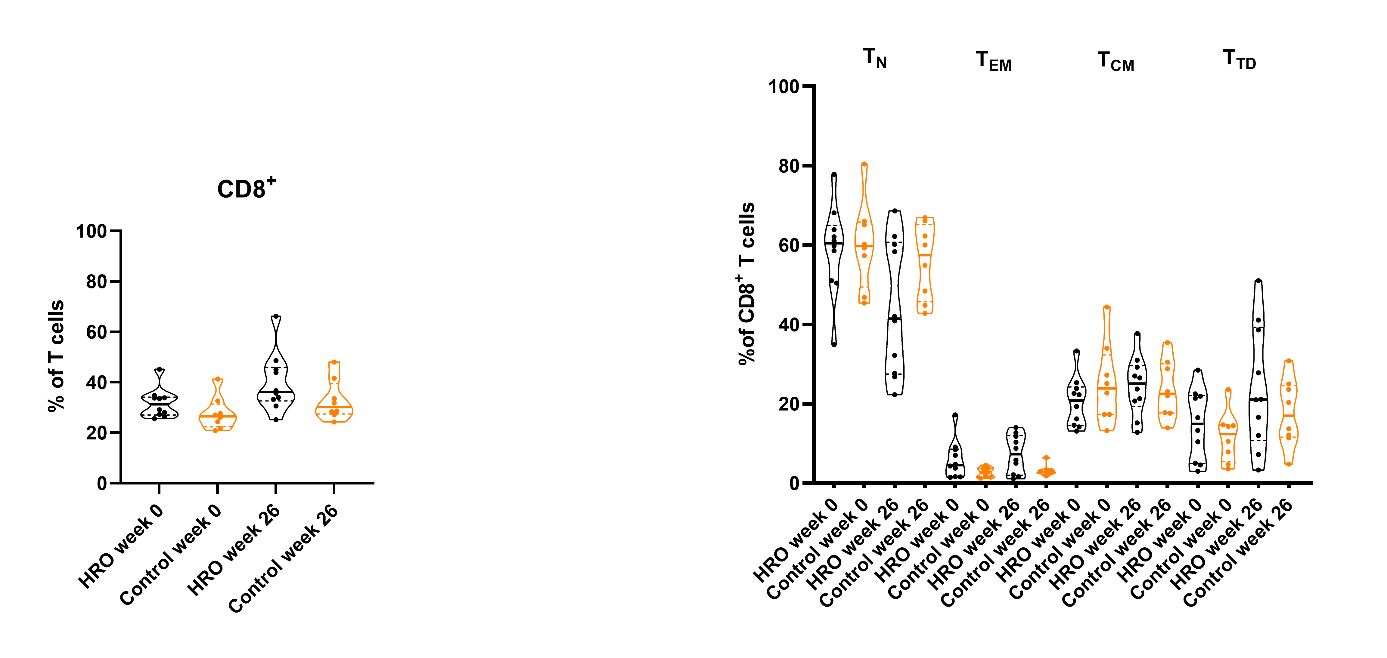


**G H**

**Suppl. Figure 2.** Frequencies of PBMC populations in investigated groups during the study period of 26 weeks. **(A)** T cells (CD14^-^CD56^-^CD3^+^), **(B)** B cells (CD3^-^ CD19^+^), **(C)** CD4^+^ naïve (T_N_), central memory (T_CM_), effector memory (T_EM_) and terminally differentiated (T_TD_) T cells, **(D)** Monocytes (CD56^-^CD3^-^CD19^-^CD14^+/-^), **(E)** NK cells (CD14^-^CD3^-^CD56^+^), **(F)** NKT-like cells (CD14^-^CD3^+^CD56^+^), and (**G**) CD8^+^ T cells and (**H**) CD8^+^ T cell subpopulations, compared between patients with psoriasis on HRO treatment (n=10), and patients with psoriasis on placebo (n=8). Each symbol represents one individual. Violin plot (truncated) shows median and quartiles. Mann-Whitney test was used to compare abundance of subsets of PBMCs in both groups. Differences were considered statistically significant for p ≤ 0.05, indicated as * ≤ 0.05 and ** ≤ 0.01.


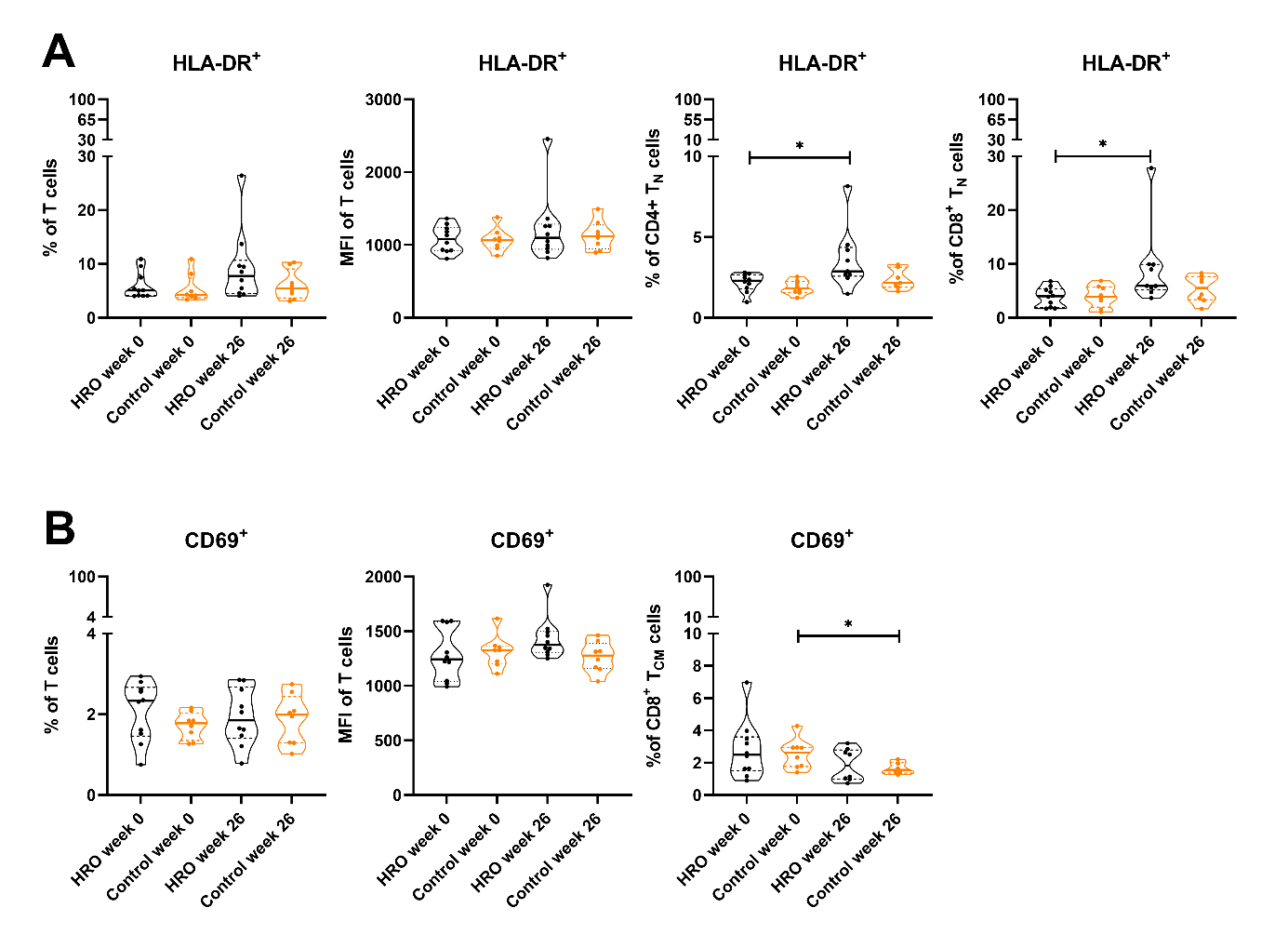


**Suppl. Figure 3**. Frequency and expression of HLA-DR (**A**) and CD69 (**B**) cell surface marker in T cells and subsets in both groups, HRO and Control. Each symbol represents one individual. Violin plot (truncated) shows median and quartiles. Mann-Whitney test was used to compare abundance of subsets of PBMCs in study groups. Differences were considered statistically significant for p ≤ 0.05, indicated as * ≤ 0.05.


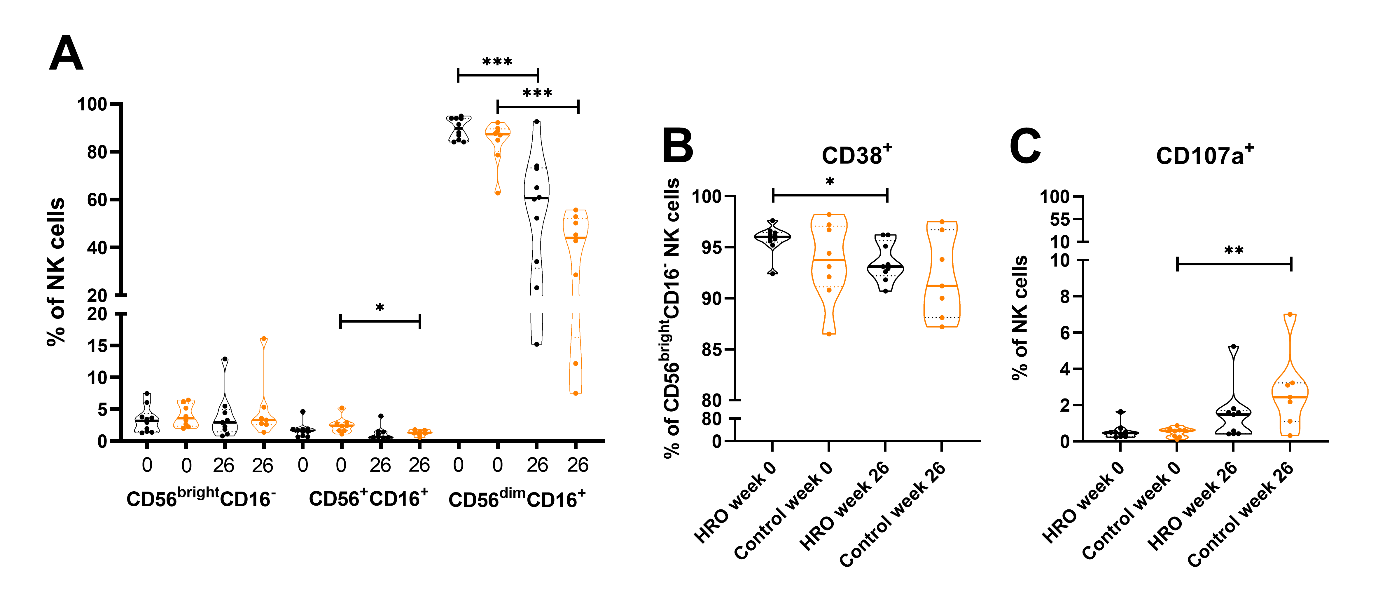


**Suppl. Figure 4**. Frequency of CD107a in NK cells in investigated groups during the study period of 26 weeks. The data represents HRO patients and Control measured in different time points (week 0 and 26). Each symbol represents one individual. Violin plot (truncated) shows median and quartiles. Mann-Whitney test was used to compare abundance of subsets of PBMCs in both groups. Differences were considered statistically significant for p ≤ 0.05.
